# Experimental Study of Rubberized Concrete Stress-Strain Behavior for Improving Constitutive Models

**DOI:** 10.3390/ma11112245

**Published:** 2018-11-11

**Authors:** Kristina Strukar, Tanja Kalman Šipoš, Tihomir Dokšanović, Hugo Rodrigues

**Affiliations:** 1 Faculty of Civil Engineering and Architecture Osijek, Department for Technical Mechanics, Vladimira Preloga 3, Osijek HR-31000, Croatia; tkalman@gfos.hr; 2 Faculty of Civil Engineering and Architecture Osijek, Department for Materials and Structures, Vladimira Preloga 3, Osijek HR-31000, Croatia; tdoksanovic@gfos.hr; 3 RISCO—School of Technology and Management, Polytechnic Institute of Leiria, Rua General Norton de Matos, Apartado 4133, 2411-901 Leiria, Portugal; hugo.f.rodrigues@ipleiria.pt

**Keywords:** rubberized concrete, rubber content, constitutive model, ductility, energy absorption

## Abstract

Inclusion of rubber into concrete changes its behavior and the established shape of its stress-strain curve. Existing constitutive stress-strain models for concrete are not valid in case of rubberized concrete, and currently available modified models require additional validation on a larger database of experimental results, with a wider set of influential parameters. By executing uniaxial compressive tests on concrete with rubber substituting 10%, 20%, 30%, and 40% of aggregate, it was possible to study and evaluate the influence of rubber content on its mechanical behavior. The stress-strain curve was investigated in its entirety, including compressive strength, elastic modulus, strains at significant levels of stress, and failure patterns. Experimental results indicated that increase of rubber content linearly decreases compressive strength and elastic modulus, but increases ductility. By comparing experimental stress-strain curves with those plotted using available constitutive stress-strain models it was concluded that they are inadequate for rubberized concrete with high rubber content. Based on determined deviations an improvement of an existing model was proposed, which provides better agreement with experimental curves. Obtained research results enabled important insights into correlations between rubber content and changes of the stress-strain curve required when utilizing nonlinear material properties.

## 1. Introduction

Structural concrete is a non-linear material both at ultimate strength limit states and service loads, as well as for certain design situations and analysis of complex problems, which implies that linear analysis may not be sufficient [1]. Being that concrete is a complex composite material its constitutive stress-strain relationship depends on its mix design, material composition, and internal microscopic structure. It is usually considered to be a brittle material that tends to fracture without significant deformations, which is associated to its low ductility [2].

In order to improve ductility of reinforced concrete elements, the concrete’s core is usually confined with steel hoops (spirals or stirrups). Nowadays there are additional ways to improve the ductility of concrete, which take into consideration sustainability and environmental pollution. One such method is the substitution of natural aggregates with recycled tire rubber in various forms: powder, crumb or chipped rubber particles [3,4,5]. The ductility and other mechanical properties of such rubberized concrete can be described with a complete stress-strain curve, which is necessary for rational design and non-linear analysis of structural elements.

Until today, stress-strain curves were obtained mostly for normal, lightweight [6], high-strength [7,8,9,10], and high-performance [11,12,13] concrete. The stress-strain curve of concrete consists of an ascending branch up to the peak stress and a descending branch until total fracture, which makes the process of generating a stress-strain curve complicated due to a large number of its shape influence parameters. These parameters include both testing conditions (specimen size, strain or stress rate, gauge length, etc.) and concrete characteristics (w/c ratio, characteristics and content of cement and aggregate, etc.). As there is no universal standard for determining the compressive stress-strain behavior of test specimens, parameters of such a test are often varied in executed research campaigns. Among the first analytical models to completely describe experimentally obtained stress-strain curves were those proposed by Hognestad [14], Kent and Park [15], Mander et al. [16], Wang et al. [6], Carreira and Chu [10], and CEB [17]. Their models were later used as a base for future studies of stress-strain curves of concrete with different admixtures, including rubber [3,4,18,19,20,21,22,23,24,25,26].

Inclusion of rubber into concrete to replace a certain percentage of aggregate can change its mechanical properties and consequently influence the shape of the stress-strain curve, compared to commonly used concrete. Due to induced changes, available constitutive stress-strain models for concrete are not valid in case of rubberized concrete, and available modified models need to be experimentally verified on more compositions. Considering specific aspects of rubberized concrete, this paper will present previous studies of rubberized concrete’s behavior, new and literary available experimental investigations, and stress-strain curve analytical models. The main contribution of this paper is based on the applicable constitutive compressive axial model and direct guidelines of utilization of rubberized concrete in structural concrete elements based on values of compressive strength and deformations.

## 2. Literature Survey

### 2.1. Experimental Characterization

Previous investigations of rubberized concrete were mostly focused on durability and mechanical properties of rubberized concrete. It was concluded that with the increase of rubber content in concrete mixes, compressive strength and elastic modulus decrease, and strain at fracture increases [27,28,29]. However, there are not enough investigations of the effect of rubber particles on the uniaxial compressive stress-strain behavior of rubberized concrete.

Compressive stress-strain behavior of rubberized concrete with 12.5%, 25.0%, 37.5%, and 50.0% was investigated by Khaloo et al. [4], to determine the influence of replacing fine (F) and coarse (C) aggregate with crumb and chipped rubber, respectively. They executed compressive displacement controlled tests of hardened concrete cylindrical specimens to obtain stress-strain curves, utilizing a speed of 0.005 mm/s. The curves indicated that the behavior of rubberized concrete was more nonlinear compared to that of plain concrete (P) [4], which might have been accounted to lower compressive strength of these mixes. A comparison between investigated mixes revealed that the behavior was more nonlinear for mixes where coarse aggregate is substituted with chipped rubber than for mixes where fine or both fine and coarse aggregate were replaced with crumb and chipped rubber.

L. Li et al. [18] performed an investigation on low-volume rubberized concrete with five different rubber volume content levels (2%, 4%, 6%, 8%, 10%) and five different particle sizes (0.173 mm, 0.221 mm, 0.535 mm, 2 mm, and 4 mm). The loading process was controlled by displacement with a loading speed of 0.003 mm/s. By comparing peak and ultimate strains researchers concluded that the ultimate strain of rubberized concrete was higher for larger rubber content and smaller particle size. The capability of crack prevention and plastic deformation was higher as small rubber particles were not only distributed on the interface between the aggregate and the cement matrix, but also scattered in the interface within the cement matrix. On the contrary, larger rubber particles were mainly in the interface between aggregates and cement matrix, thus having almost no influence on the deformation of the cement matrix, which led to the concentration of plastic strain in concrete.

Xie et al. [19] substituted 4%, 8%, 12%, and 16% volume of sand with crumb rubber (CR), added 1% of steel fibers (SFs) by volume and replaced 100% of natural coarse aggregate (NCA) with recycled coarse aggregate (RCA) in concrete mixes. Thus two reference mixes were produced, one with NCA (marked with * in Table 1) and the other with 100% replacement of NCA with RCA. Axial load was applied at a displacement rate of 0.003 mm/s. It was observed that 100% replacement of NCA by RCA resulted in larger strain at peak stress and a smoother and straighter descending branch of the stress-strain curve. It was concluded that rubberized steel fiber recycled aggregate concrete (RSFRAC) has greater ductility than NCA concrete, although RSFRAC exhibited a reduced strength and stiffness. The effect of rubber content in the stiffness of RSFRAC was not clear before the peak stress but became apparent after the peak stress.

**Figure 1:**
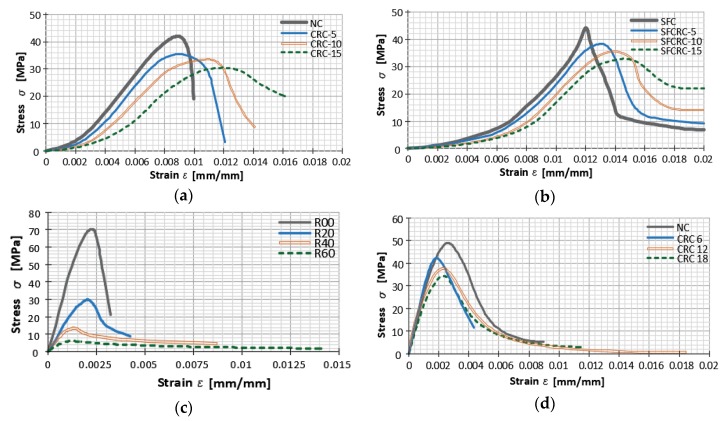
Stress-strain curves of: (**a**) normal concrete (NC), rubberized concrete with 5% rubber (CRC-5), 10% rubber (CRC-10), 15% rubber (CRC-15) [25]; (**b**) steel fiber concrete (SFC), steel fiber rubberized concrete with 5% rubber (SFCRC-5), 10% rubber (SFCRC-10), 15% rubber (SFCRC-15%) [25]; (**c**) traditional concrete (R00), rubberized concrete with 20% rubber (R20), 40% rubber (R40), and 60% rubber (R60) [3]; (**d**) NC, concrete with 6% crumb rubber (CRC6), 12% (CRC12), 18% (CRC18) [23].

Noaman et al. [25] conducted a similar investigation on the compression toughness of rubberized concrete (RC) with 5%, 10%, and 15% crumb rubber, and rubberized steel fiber concrete (RSFC) with 0.5% steel fibers amounts of crumb rubber as in RC. Cylindrical specimens of both mixtures were subjected to an axial load rate of 0.3 MPa/s, after 28 days of curing. Researchers came to the conclusion that the addition of rubber leads to the increase in ductility and strain capacity as shown in Figure 1a,b. Additionally, it can be seen that steel fibers had an impressively larger effect on stress and increase in capacity in comparison with RC.

D.V. Bompa et al. [3] investigated the compressive stress-strain response of a traditional concrete (R00) compared to the response of concretes with 20% (R20), 40% (R40), and 60% (R60) of rubber aggregate. To determine the complete stress-strain behavior including the post peak response, cylindrical specimens were tested under uniaxial compression utilizing displacement control at a rate of 0.001 mm/s. Recorded average stress-strain curves are presented in Figure 1c [3]. The stress-strain curves included both pre-peak and post-peak behavior as recorded in tests. The pre-peak behavior of the concrete tests was strongly influenced by the rubber replacement of the mineral aggregates.

Aslani et al.’s [21,22] study investigated the effect of crumb rubber on the mechanical properties of self-compacting rubberized concrete (SCRC) with the addition of chemical admixtures (fly ash, slag, and silica fumes) and pre-treated rubber. In three different concrete mixes they replaced 10%, 20%, 30%, and 40% fine natural aggregates with crumb rubber (CR) of 2 mm and 5 mm size, and coarse natural aggregates with crumb rubber of 10 mm size. Cylindrical specimens were tested at 28 days and loaded under compression until failure to obtain the stress-strain behavior. Axial loads were applied at a displacement rate of 0.003 mm/s. As the percentage of rubber increased for each of three SCRC series, the overall peak strain decreased. Results also indicated that higher strains were generated at lower stress as the percentage of rubber replacement was increased. However, results of this investigation were not conclusive due to the missing descending portion of curves.

D. Li et al. [23] investigated compressive stress-strain behavior of normal concrete (NC) and crumb rubber concrete (CRC) with 6%, 12%, and 18% crumb rubber (CR) aggregates. They tested three unconfined cylindrical specimens for each concrete mix under uniaxial displacement-controlled compression loading at a rate of 0.001 mm/s. Test results of the experiment are shown in Figure 1d where it can be seen that the initial part of the curve is linear, after which stiffness reduces up to the peak stress. At higher strains, the descending portion tended to reach a constant stress level.

A summary of described previous research is presented in Table 1 from which a conclusion can be made that replacement of natural fine and coarse aggregates with rubber allows a more uniform crack development and enables slower crack propagation. Considering the stress-strain curves, rubberized concrete specimens exhibited larger deformations compared to plain concrete specimens under same loading conditions. Quasi-plastic behavior of rubberized concrete is noticeable on the post-cracking part of the stress strain curve with small change in deformation without losing load bearing capability. Hence, obtained curves support the assertion that rubber particle usage in concrete results in concrete failure with larger deformations and higher energy dissipation.

### 2.2. Uniaxial Stress-Strain Constitutive Models

**Figure 2:**
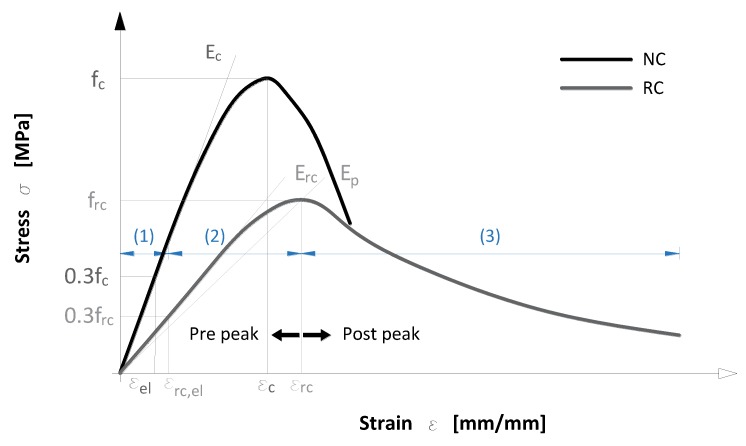
Stress-strain curve which describes mechanical parameters needed for establishing constitutive model of rubberized concrete.

To mathematically simulate concrete’s behavior under uniaxial load, constitutive uniaxial models were developed. It should be noted that there are also other constitutive models which describe the behavior of concrete under various stress states, and for various purposes [30], but due to lack of experimental data have not been developed for rubberized concrete. Experimental results show that differences in concrete’s mixture proportions, additives, etc., affect concrete behavior, namely the shape of the uniaxial curve. Thus, one constitutive model cannot fit all concretes, and for rubberized concrete only a few have been proposed. These models take into account rubber content and size of rubber particle, which can be seen in a condensed form in Table 2. To obtain a stress-strain curve as in Figure 2, input data in models are peak compressive stress $f_{c}$, peak strain $\varepsilon_{c}$ and elastic modulus $E_{c}$ of normal concrete (NC) which are used to calculate peak compressive stress $f_{rc}$, peak strain $\varepsilon_{rc}$ and elastic modulus $E_{rc}$ of rubberized concrete (RC). However, researchers considered different parameters while developing constitutive models described with a stress factor $\sigma /f_{rc}$.

L. Li et al. [18] presented an improved constitutive model based on the one given in the Chinese design code for concrete structures (GB50010-2002) [31]. To improve the existing model, they considered different rubber mixture methods, absolute value of rubber content ρ, rubber particles size d, sand rate reduction factor k and concrete’s compressive strength. With constitutive parameters α and β, ascending and descending parts of the stress-strain curve could be obtained. Aslani [20] developed a stress-strain relationship for rubberized concrete based on Aslani and Nejadi’s [32] model and an experimental results database from several studies. Based on these studies he proposed different coefficients for peak compressive stress (α, β) and elastic modulus of elasticity (φ, ψ). In his proposed model he used modified material parameters $\rho_{m,a}$ and $\rho_{m,d}$ for obtaining ascending and descending parts of the stress-strain curve, and two coefficients of linear equation  φ and κ, which D. Li et al. [23] later modified. Bompa et al. [3] presented a constitutive model for rubberized concrete which uses equations that they proposed to estimate the elastic modulus and peak compressive stress of rubberized concrete. The constitutive model considers the volumetric rubber ratio $\rho_{vr}$ up to 0.65 (65% of volumetric rubber replacement with mineral aggregate) and size of replaced mineral aggregate $d_{g,repl}$ with factor λ. The constitutive model has three parts; first up to the proportionally limit $\varepsilon_{rc,\;el}$ (1), second up to the peak strain $\varepsilon_{rc}$ (ascending part of the curve) (2), and third after the peak strain (descending part of the curve) (3) which depends on the post-peak crushing energy $g_{c,2}$.

## 3. Materials and Methods

To investigate compressive strength, elastic modulus and compressive stress-strain behavior of rubberized concrete compared to normal concrete (NC), in total 30 cubic (150 × 150 × 150 mm^3^) and 60 cylindrical (∅150 mm × 300 mm) concrete specimens were casted out of 10 different concrete mixes and tested after 28 days.

### 3.1. Materials

**Figure 3:**
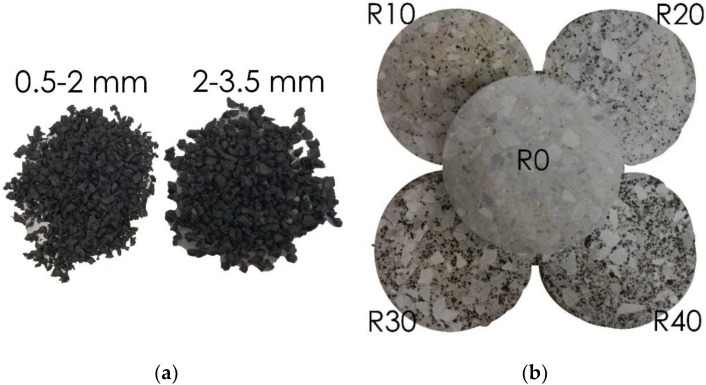
(**a**) Particles of recycled rubber aggregate used in concrete mixes; (**b**) crosssections of cylindrical specimens for different percentages of rubberized aggregate.

**Figure 4:**
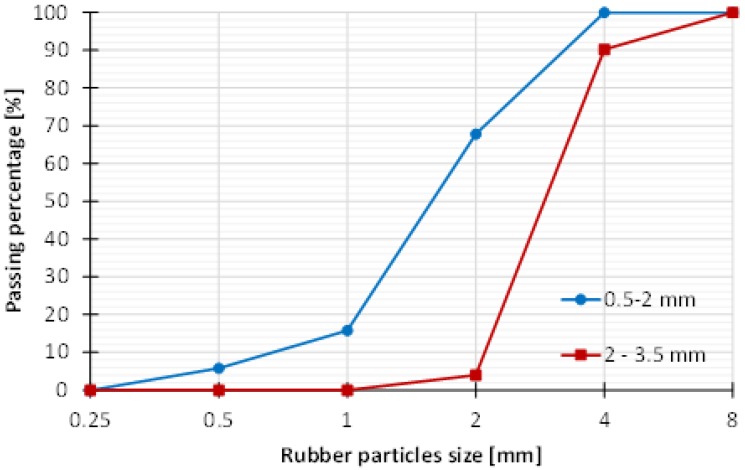
Particle size distribution of crumb rubber aggregate.

Materials used for production of concrete mixes include Portland cement 32.5 N, which conforms to HRN EN 197-1:2005, and mineral (MA) and recycled aggregates (RA). Mineral aggregates were sand of 0–2 mm and 2–4 mm fraction, and coarse aggregates which included gravel of 4–8 mm and 8–16 mm fraction. Recycled aggregate crumb rubber (CR) with particles of 0.5–4 mm size and density of 1050 kg/m^3^ was used in concrete mixes as substitute for 10%, 20%, 30%, and 40% ratio of the volume of sand. Shape of crumb rubber particles was irregular as it can be seen in Figure 3. Particle size distribution of crumb rubber aggregate is given in Figure 4.

### 3.2. Mix Designs

Concrete mixes are labeled with NC-R*x*-*y* where R represents rubber, *x* refers to the percentage of crumb rubber in mix (0–40%), and *y* to the amount of cement in mix (400 or 500 kg/m^3^).

Two series of concrete mixes were made with a principal differentiation in the amount of used cement (Table 3)—Series I with 400 kg/m^3^ of cement and Series II with 500 kg/m^3^ of cement. In each concrete mix, water to cement (w/c) factor was constant. In Series I, normal concrete had a composition of 323.6 kg/m^3^ sand, total of 1473.4 kg/m^3^ gravel of different fractions, 180 kg/m^3^ of tap water, and 2 kg/m^3^ of super-plasticizer Glenium (GL) ACE 430 to obtain better workability. Additional water was necessary due to the dry surface of natural aggregates. Other rubberized concrete mixes in this group contained rubber which replaced 10–40% of volume of sand. Quantities of rubber used were 66.8 kg/m^3^, 133.6 kg/m^3^, 200.6 kg/m^3^, and 266.2 kg/m^3^ for ratio of 10%, 20%, 30%, and 40% of crumb rubber, respectively. In mix NC-40-400, 4 kg/m^3^ of super-plasticizes Glenium ACE 430 was needed and 0.8 kg/m^3^ of RheoMATRIX (RM) to enable the right balance between fluidity, passing ability, and resistance to segregation. In Series II, normal concrete mix without rubber was composed of 281 kg/m^3^ sand, 1277 kg/m^3^ gravel of different fractions, 198.1 kg/m^3^ tap water, and 2.6 kg/m^3^ Glenium ACE 430. Mixtures with rubber differentiated as 26.9 kg/m^3^ of water was added and the amount of CR, sand and gravel of 0–4 mm fraction was varied. Quantities of CR used were 58.4 kg/m^3^, 116.8 kg/m^3^, 175.2 kg/m^3^, and 233.8 kg/m^3^ for ratio of 10%, 20%, 30%, and 40% of CR, respectively.

Fresh properties of concrete were investigated according to EN 12350-2:2009 [33], to EN 12350-6:2009 [34], and to EN 12350-7:2009 [35] for slump test, density and porosity, respectively. Results presented in Table 4 showed that the mass and density of rubberized concrete are decreased, when compared with the reference concrete, and porosity is increased with increased crumb rubber content.

### 3.3. Specimen Preparation and Testing Arrangement

According to HRN EN 12390-1:2012 [36] guidelines for shape, dimensions of specimens and moulds, and HRN EN 12390-2 [37] guidelines for making and curing specimens, both cubic (150 × 150 × 150 mm^3^) and cylindrical (∅150 mm × 300 mm) specimens were casted for each mix of both series. The cylinders and cubes were demolded 24 h after casting and then continuously moist-cured for 28 days. After this period, test specimens were measured and prepared for testing.

**Figure 5:**
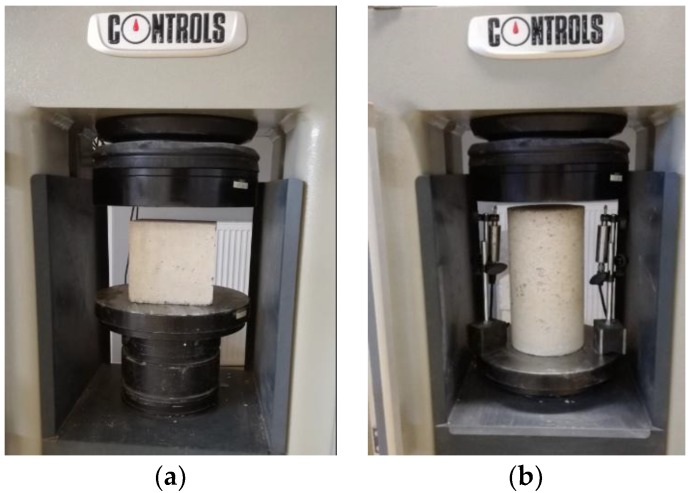
(**a**) Compressive strength testing arrangement; (**b**) stress-strain behavior testing arrangement.

Compressive strength, modulus of elasticity, and stress-strain curves were tested under uniaxial compression in a four-column high stiffness welded frame Controls Automatic Compression machine which conforms to HRN EN 12390-4:2000 [38], with a capacity of 2000 kN, which was connected to a computer for data logging (Figure 5a). Compressive strength was tested on 30 cubic concrete test specimens, three specimens from each mix given in Table 3, in accordance with HRN EN 12390-3:2001 [39]. Elastic modulus was tested on 30 cylindrical concrete specimens following HRN EN 12390-13:2013 [40] guidelines. To obtain stress-strain curves, samples were tested in a Controls Automatic Compression machine on cylindrical specimens. Tests were carried out using stress control with a rate of 0.01 MPa/s. Samples were placed on the bottom plate of the testing machine. To track axial pre-peak and post-peak deformations of concrete cylindrical specimens, two Controls Linear Variable Differential Transformers (LVDT) with a gauge length of 10 mm were placed next to the specimen, between bottom and upper plates (Figure 5b). As the upper plate is movable, LVDT measured its displacement which is the same as the axial deformation of test specimen. Test was controlled via computer software E-module by Controls, which also recorded obtained results, i.e., stress-strain curves.

## 4. Test Results and Data Analysis

### 4.1. Compressive Strength

**Figure 6:**
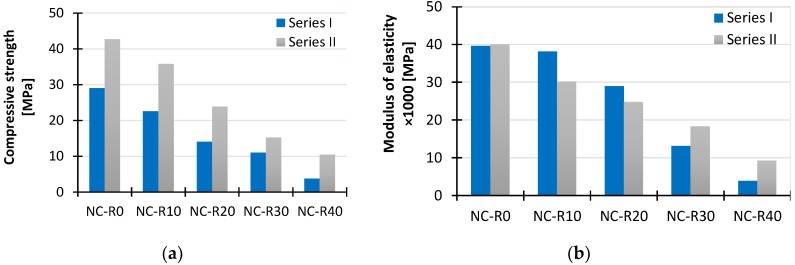
(**a**) Compressive strength test results on cubic specimens; (**b**) elastic modulus test results on cylindrical specimens.

**Figure 7:**
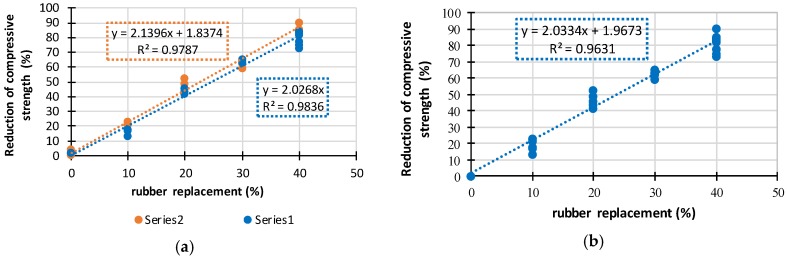
(**a**) Reduction of compressive strength depending on the cement amount; (**b**) reduction of compressive strength depending on the crumb rubber content.

Results obtained from tests that include mean values (mean), standard deviation (SD) and coefficient of variation (CoV) are given in Table 5. It can be seen that the ultimate compressive strength reduces with increase in CR content for both concrete series. Compressive strength of concrete mixes (Series I) with 10%, 20%, 30%, and 40% CR content was reduced by 22%, 52%, 62%, and 87%, respectively, compared to the compressive strength of reference mix NC-0-400. Compressive strength of concrete mixes (Series II) with 10%, 20%, 30%, and 40% CR content was reduced by 16%, 44%, 67%, and 76%, respectively, compared to the compressive strength of reference mix NC-0-500. By comparing compressive strengths of samples with the same amount of CR, in Figure 6a, it can be concluded that a higher amount of cement (500 kg/m^3^) in Series II resulted in lower reductions of strength and general in higher strength. Figure 7a,b shows that the reduction of compressive strength with increase of CR content is linear.

### 4.2. Elastic Modulus

In Table 5 are given mean values of elastic modulus test results where it can be seen that this property is reduced with increase of CR content, as expected. Elastic modulus of concrete mixes (Series I) with 10%, 20%, 30%, and 40% CR content was reduced by 4%, 27%, 67%, and 90% respectively, compared to the elastic modulus of reference mix NC-0-400. Elastic modulus of concrete mixes (Series II) with 10%, 20%, 30%, and 40% CR content was reduced by 25%, 38%, 54%, and 77% respectively, compared to the elastic modulus of reference mix RNC-0-500. By comparing results of concrete mixtures from both series, in Figure 6b, it can be concluded that the reduction of elastic modulus is lower for concrete mixtures with 10% and 20% CR content from Series I. However, for higher CR amount in concrete mixes, 30% and 40%, reduction is lower for the ones from Series II.

### 4.3. Stress-Strain Behavior

**Figure 8:**
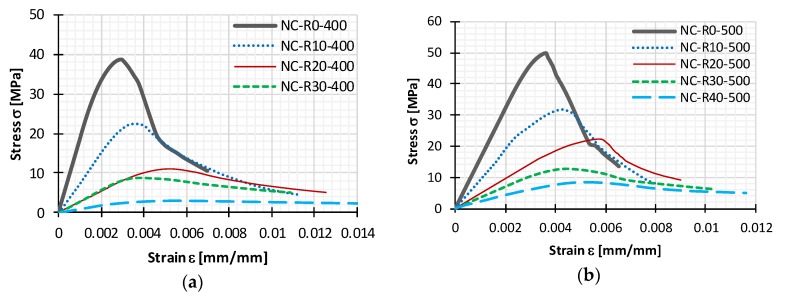
Compressive stress-strain curves for reference normal concrete (NC) and rubberized concrete (RC) with 10%, 20%, 30%, and 40% crumb rubber from (**a**) Series I and (**b**) Series II.

The compressive stress-strain behavior of cylindrical test specimens is presented with curves in Figure 8a for test specimens of Series I, and in Figure 8b for test specimens of Series II. Curves for both series are as average for each mix, which clearly depicts that all stress-strain curves from different mixes follow a similar trend.

Stress-strain curves were divided in the pre-peak and post-peak behavior as shown in Figure 2. Pre-peak behavior is similar for both reference NC mixes and RC mixes, being mostly linear with differences in stiffness and curvature shape near peak stress. Stiffness is greater and curvature smaller for reference NC mixes, with a reverse proportional tendency with increase of CR content. These differences can be seen in Figure 2, where two characteristic curves are presented, one for normal and the other for rubberized concrete. The post-peak behavior presents other differences. For reference mixes, the softening branch has a high reduction of stiffness, when compared with rubberized concrete. With increased CR content this post peak behavior has an increase in the softening stiffness leading to a higher ultimate strain. Additionally, in Figure 8 it can be noticed that some overlapping of curves occurs at higher strains, which can be attributed to a similar mechanism of failure (exhaustion of tension strength perpendicular to compression), but this mechanism deteriorates more quickly with lower rubber content. The point by which indicated regions of the stress strain curves are separated, the peak stress, is reduced with increased CR content.

When comparing stress-strain curves for both series, in terms of ductility and predictability better results were obtained for Series II. Namely, with the increase in rubber content, peak stress reduction was gradual, and similar can be observed for increase in ultimate strain. Curves obtained for Series I mixes with 20% and 30% of CR content showed no difference in peak stress, with ultimate strain being greater for mix with 20% rubber than that of mix with 30% rubber. Taking into account all obtained results it can be concluded that rubber greatly influences the behavior of concrete.

### 4.4. Failure Pattern

**Figure 9:**
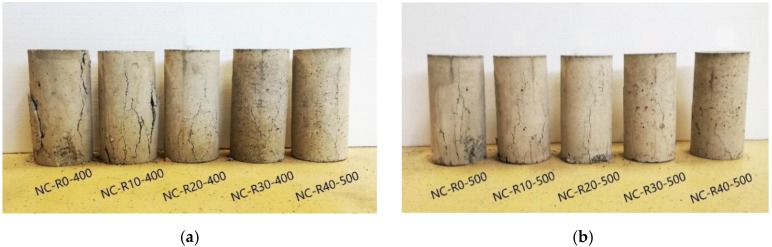
(**a**) Failure types of test specimens from Series I and (**b**) Series II.

An additional intention of performed compression tests was to observe failure patterns of cylinder specimens during loading and failure, as that can help to understand the overall behavior of specimens. Figure 9a,b show specimens of each mix after failure.

Figures illustrate that reference NC mixes fail in a more brittle manner, suddenly and without any indications after reaching peak stress. Specimens were separated into large pieces and cracks were wide. However, mixes with rubber fail with less pronounced cracks, in more uniform manner, which is in accordance with previously analyzed strain-strain curves, i.e., post peak behavior. Namely, after reaching peak stress test specimens could withstand further increase of strain without sudden loss of force, similar to observations made by Khaloo et al. [4]. Specimens did not break into larger pieces, mainly due to bridging of cracks by rubber particles. The number of cracks increased but they were narrower and created a fine mesh. Xie et al. [19] and D. Li et al. [23] attributed this more progressive and uniform development of micro cracks at interfaces to weak interfacial transition zone (ITZ) between rubber particles and cement matrix, which has a good ability to restrain compressive deformations and allow strain increase at a higher rate than in normal concrete. Cracks are restrained from emergence and development, and the fracture of concrete is alleviated to some degree.

## 5. Application and Improvement of Existing Constitutive Models

**Figure 10:**
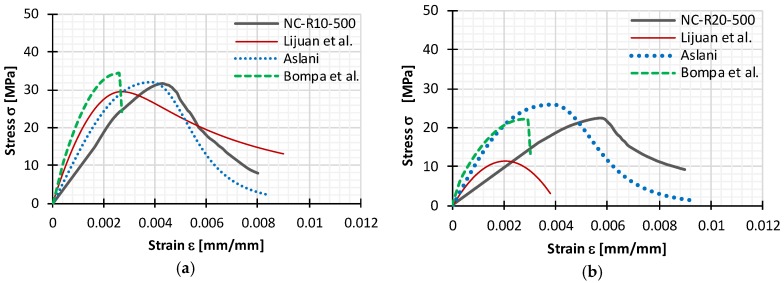

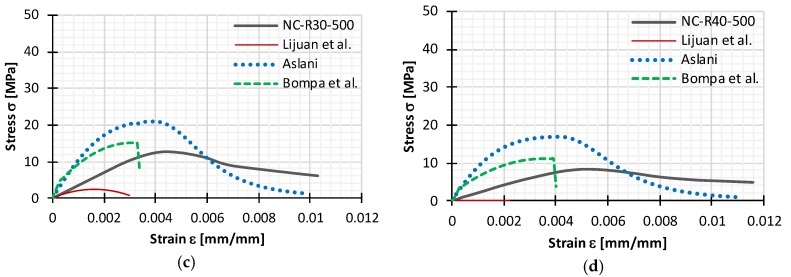
Comparison of experimental stress-strain curves for (**a**) NC-R10-500; (**b**) NC-R20-500; (**c**) NC-R30-500; and (**d**) NC-R40-500 with existing constitutive models.

Constitutive models for rubberized concrete presented in Section 2.2 were each used to describe experimentally obtained stress-strain curves for Series II. The comparison of stress-strain curves is shown in Figure 10, from which it can be observed that available models could not accurately predict experimentally determined behavior of rubberized concrete—higher elastic modulus values, lower values of peak strain, unsuitable peak compressive stresses, and overall shape of the curve. Values of peak stress for rubberized concrete $f_{rc\;}$ and corresponding peak strain $\varepsilon_{rc}$, obtained experimentally and from constitutive models, are presented in Table 6.

L. Li et al.’s [18] model predicts significantly lower peak compressive stress values. Additionally, instead of increasing peak strain with increasing rubber content, strain is decreasing, which is probably due to the fact that their model was developed for lower amounts of rubber (up to 12%). Bompa et al.’s [3] model was developed for higher rubber content, and thus provides a better prediction of the peak compressive stress, but peak strain and shape of the curve are inadequate. Aslani’s [21] model can describe the shape of the curve well, with both ascending and descending branches, but peak stress and strain are higher and lower, respectively. However, because the curve shape is adequate, his constitutive model could be improved with slight changes as follows:

- As Bompa’s expression for peak compressive stress $f_{rc}$ takes into consideration higher rubber amount $\rho_{vr}$ and size of replaced mineral aggregate particles with factor λ, it is adopted for its estimation.
- To take into consideration some of the influential parameters ($\rho_{vr}$ and λ) for obtaining peak compressive stress $f_{rc}$ and for a more accurate description of the ascending part of the stress-strain curve, a new expression for the elastic modulus of rubberized concrete $E_{rc}$ is developed. This expression takes into account compressive strength $f_{c}$ obtained on cubic specimens of normal concrete.
- Coefficients that are used for calculating compressive stress and elastic modulus (α, β, φ, ψ) are excluded from equations.
- The Constitutive model is now a single model capable of predicting stress-strain response from the origin to ultimate strain, which is expressed with Equations (1)–(9).

Input parameters are compressive strength $f_{c}$ obtained on reference normal concrete cubic specimens, proportionally limit $\varepsilon_{el}$ at 0.4 $f_{c}$ [41] obtained from a stress-strain curve of normal concrete without rubber aggregate, relative volumetric rubber ratio $\rho_{vr}$, and size of replaced mineral aggregate $d_{g,repl}$ considered with factor λ. With known input parameters, it is possible to calculate:

Peak compressive stress for rubberized concrete:

(1a) $$\;f_{rc}=\frac{1}{1+2\cdot {\left(\frac{3\lambda \rho_{vr}}{2}\right)}^{3/2}}f_{c}\;$$

(1b) $$\;\lambda =\{\begin{array}{c} 2.43\to \;d_{g,repl}\;\epsilon \;\left(0,5\right)\\ 2.90\to \;d_{g,repl}\;\epsilon \;\left(0,d_{g,max}\right)\\ 2.08\to \;\;d_{g,repl}\;\epsilon \;\left(5,d_{g,max}\right) \end{array}\;$$

Tangent elastic modulus for rubberized concrete:

(2) $$\;E_{rc}=\frac{0.4f_{c}}{\varepsilon_{el}}\cdot \exp\left(-\lambda \rho_{vr}\right)\;$$

Coefficient for calculating strain at peak stress:

(3) $$\;v=\frac{f_{rc}}{17}+0.8\;$$

Strain at peak stress for rubberized concrete:

(4) $$\;\varepsilon_{rc}=\left(f_{rc}/E_{rc}\right)\left(\frac{v}{v-1}\right)\;$$

Secant modulus of elasticity:

(5) $$\;E_{p}=f_{rc}/\varepsilon_{rc}\;$$

Coefficients of linear equation:

(6) $$\;\varphi =35\times {\left(12.4-1.66\times 10^{-2}f_{rc}\right)}^{-0.9}\;$$

(7) $$\;\kappa =0.75\exp\left(-\frac{911}{f_{c}}\right)\;$$

Modified material parameter:

(8) $$\;\rho_{m}={\left[1.02-1.17\left(E_{p}/E_{rc}\right)\right]}^{-0.74}+\left(\varphi +\kappa \right)\;;\;0<\varepsilon <\varepsilon_{u}\;$$

Stress factor to obtain the constitutive curve:

(9) $$\;\frac{\sigma }{f_{rc}}=\left[\frac{\rho_{m}\left(\varepsilon /\varepsilon_{rc}\right)}{\rho_{m}-1+{\left(\varepsilon /\varepsilon_{rc}\right)}^{\rho_{m}}}\right];0<\varepsilon <\varepsilon_{u}\;$$

**Figure 11:**
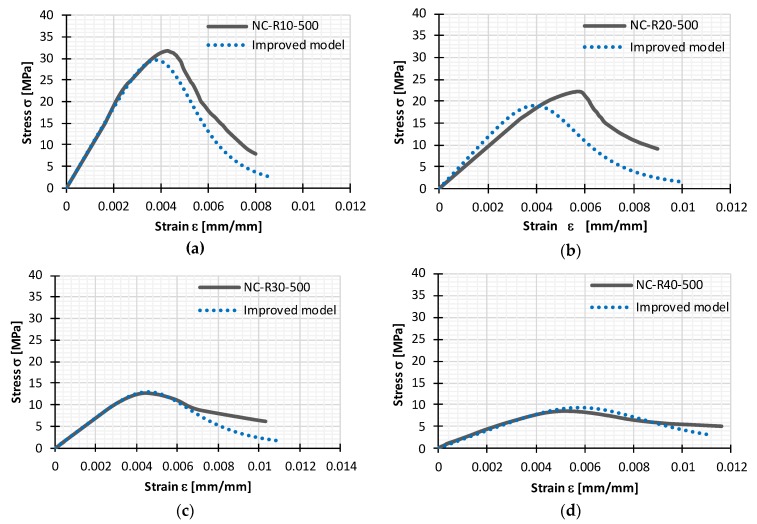
Comparison of experimental stress-strain curves for (**a**) NC-R0-500; (**b**) NC-R10-500; (**c**) NC-R30-500; and (**d**) NC-R40-500 with the improved constitutive model.

From the Table 6 it can be observed that utilization of the improved model yields estimated values that are similar to experimentally determined—peak compressive stress $f_{rc}$ is 7.3% and 15.4% lower for 10% and 20% rubber content respectively, and 1.6% and 11.1% higher for 30% and 40% rubber content respectively. Estimated peak strain for 10% and 20% rubber aggregate value was 9.1% and 27.6% lower respectively, while for 30% and 40% it was 4.4% and 11.5% higher respectively. Brackets in Table 6. reveal differences between results obtained experimentally and analytically, which are illustrated in Figure 11, where both experimental and analytical stress-strain curve are depicted.

The accuracy of improved constitutive model is analyzed with statistical performance measures presented in Table 7. Five different statistical performance measures were used in order to evaluate effectiveness constitutive model and its ability to make accurate prediction. A lower value of Mean absolute error (MAE), Root mean squared error (RMSE), Mean absolute percentage error (MAPE) and higher values of Coefficient of correlation (R) and Error € above 0.80 illustrates good efficiency and predictability of the model. Every stress-strain curve (experimental and modelled) is divided in sufficient number of sub-division in order to make effective comparison.

According to the results presented in Table 8, it is evident that the model can accurately present behaviour of rubberized concrete and its stress-strain relation. Because of the certain deviation of the model results in terms of smaller correlation coefficient and larger mean absolute percentage error related to experimental sample with 20% of rubber replacement, validation of presented model is performed.

### 5.1. Validation of Presented Constitutive Model

**Figure 12:**
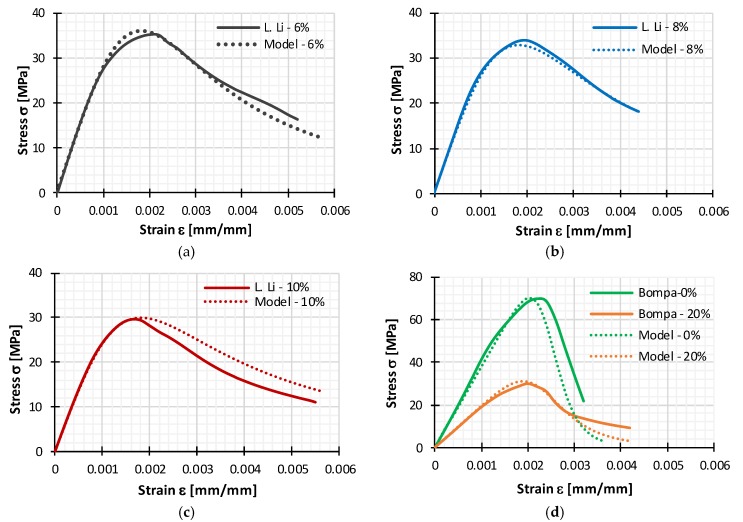
Comparison of experimental stress-strain curves for (**a**) tire rubber concrete with 6% rubber aggregate; (**b**) tire rubber concrete with 8% rubber aggregate; (**c**) tire rubber concrete with 10% rubber aggregate (data from L. Li [18] for tire rubber concrete with rubber aggregate of 2 mm size); (**d**) normal concrete with 0% rubber aggregate and for rubberized concrete with 20% rubber aggregate (data from D.V. Bompa et al. [3]).

In order to verify that a given constitutive model can be suitable for reliable results, a completely new experimental data [3,18] that were not used in evaluation of model were used for efficient validation. Samples that are used have different percentage of rubber replacement up to 20%. As it is presented in Figure 12 and Table 9, presented model can efficiently predict stress-strain relation of normal rubberized concrete. All statistical measures also approved the applicability of Constitutive model.

### 5.2. Parametric Analysis

**Figure 13:**
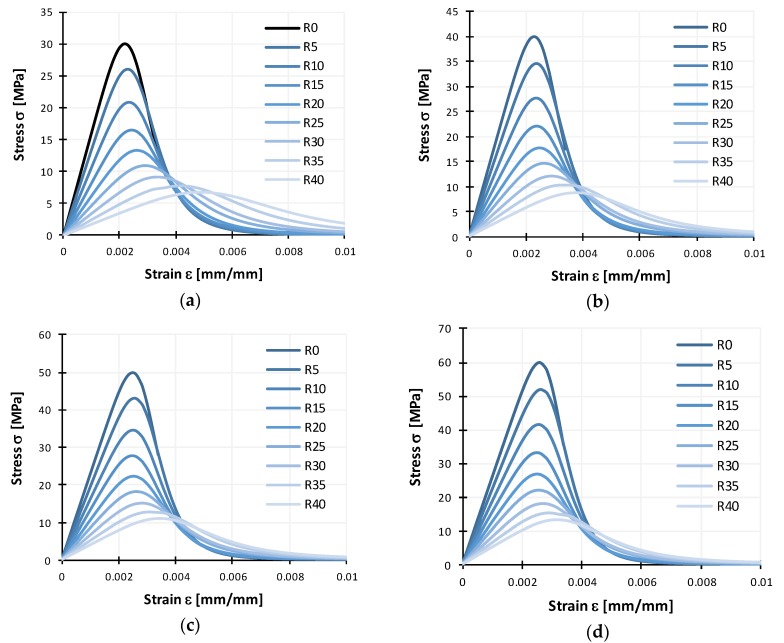
Comparison of stress-strain curves for normal concrete with initial compressive strength of (**a**) 30 MPa; (**b**) 40 MPa; (**c**) 50 MPa; (**d**) 60 MPa and rubber replacement up to 40%.

In order to present the most important highlights that can be concluded from current study, parametric analysis is performed. The parameters that were varied are initial compressive strength of normal non-rubberized concrete (from 30 MPa to 60 MPa, with increment of 10 MPa) and percentage of rubber replacement (from 5% to 40%, with increment of 10%) as it is presented in Figure 13.

**Figure 14:**
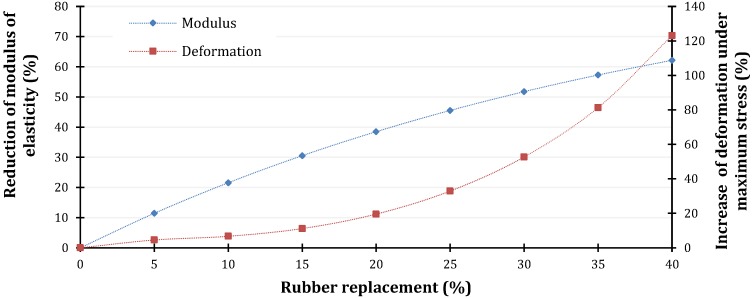
Variation of modulus of elasticity and maximum stress deformation for rubberized concrete.

According to the presented results it is evident that larger deformability of rubberized concrete is more obvious with concrete with smaller compressive strength (corresponding deformation of maximum stress is higher) with higher percentage of rubber replacement (Figure 14).

Change of rubber replacement is directly related to modulus of elasticity, with consequence of decrease up to 60% for highest rubber replacement.

**Figure 15:**
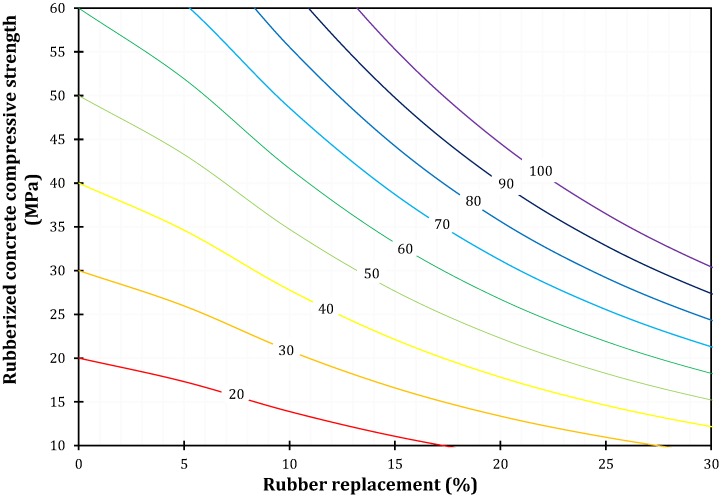
Model for rubberized concrete compressive strength.

The deficiency of any specific directions about mix design of rubberized concrete is generally absent. Therefore, from parametric analysis with large number of increments in terms of normal concrete compressive strength and rubber replacement resulted in the initial Model for mix design of rubberized concrete (Figure 15). The compressive strength of normal concrete is presented on every curve. The application of the presented model is simple. If the compressive strength of 35 MPa for rubberized concrete is needed, then several options are available: normal concrete compressive strength of 40 MPa with 5% rubber replacement, 50 MPa with 10%, 60 MPa with 13%, 70 MPa with 17%. An extrapolation of the model is possible if it is needed.

## 6. Conclusions

Previous studies of rubberized concrete have shown that mechanical properties of concrete change with the addition of rubber aggregate, i.e., elastic modulus and compressive strength are lowered. However, obtained stress-strain curves reveal benefits, such as the reduction of softening stiffness in the post-peak area and thus improvement of ductility and energy absorption.

The primary aim of the current investigation presented in this paper was to determine correlations between rubber content and compressive strength, modulus of elasticity, complete stress-strain behavior, and specimen’s failure pattern. From obtained results the following should be emphasized:

- From experimental results regarding compressive strength, it is determined that the addition of rubber aggregate reduces compressive strength, with a linear relation.
- Experimental stress-strain curves of rubberized concrete reveal that increase of rubber content decreases elastic modulus. However, strain at peak stress and ultimate strain increase, which support the premise that rubberized concrete is more ductile than normal concrete.
- Observation of specimens after executed experiments confirms the conclusion that rubberized concrete is more ductile, due to cracks being narrower and aligned in a mesh, without separation of large parts. There is no unexpected failure of specimens, as they stay compact even after peak stress.
- Available constitutive stress-strain models were not able to adequately represent experimentally obtained stress-strain curves. One of the models was modified to encompass important parameters, so an improved constitutive model is proposed which can be used for estimation of rubberized concrete behavior. However, due to the limited number of test specimens, validation of the proposed analytical model by results from different studies is done in order to approve its accuracy.
- New model for evaluation of compressive strength of rubberized concrete, based on compressive strength of normal concrete and percentage of rubber replacement is provided.

## Figures and Tables

**Table 1:** Previous investigation on stress-strain behavior of rubberized concrete.

Author	Rubber Particles Size [mm]	Rubber Content [%]	Admixtures/Treatment	Specimen Dimensions [mm]	Loading Rate	Average Peak Stress [MPa]	Deformation at Peak Stress ×10^−3^ [mm/mm]	Max Deformation ×10^−3^ [mm/mm]
Khaloo et al. [4]	2.0–15.0	0	-	∅150 × 300	0.005 mm/s	32	6.5	25
		12.5				6.5	7.1	50
		25				1.25	18	100
		37.5				0.7	39	140
		50				0.45	62	160
L. Li et al. [18]	0.173–4.0	0	-	150 × 150 × 150	0.003 mm/s	43	2	4
		2				39	2.1	5.15
		4				36.5	2.15	5.08
		6				35	2	5.08
		8				32	1.9	5
		10				29	1.8	5.5
Xie et al. [19]	0.85–1.4	0 *	1% steel fibers 32 mm by volume	∅150 × 300	0.003 mm/s	73	2	10
		0				51	3.8	7
		4				48	3.9	16
		8				47	3.6	16
		12				45	3.7	15.6
		16				41	3.7	16
Noaman et al. [25]	1.18–2.36	0	-	∅100 × 200	0.300 MPa/s	41	8.7	9.7
		5				35	8.9	12
		10				32	11	14
		15				29	12	16
	1.18–2.36	0	0.5% steel fibers 60 mm by volume	∅100 × 200	0.300 MPa/s	43	12	14
		5				38	13.2	16
		10				35	14.2	17.7
		15				32	15	18.2
D.V. Bompa et al. [3]	0.0–10.0	0	Silica fume, fly ash	∅100 × 200	0.001 mm/s	70	2.2	3
		20				29	2.03	4.4
		40				13.2	1.32	9
		60				6.2	1.17	15
D. Li et al. [23]	1.18–2.36	0	Rubber pre-treated with NaOH and precoated with cement	∅100 × 200	0.001 mm/s	50	2.59	9.7
		6				45	1.85	4.2
		12				39	2.3	18
		18				34	2.35	11
* Reference concrete mix with natural coarse aggregate (NCA) and 0% rubber aggregate.								

**Table 2:** Constitutive models for rubberized concrete.

Authors	Constitutive Models
L. Li et al. [18]	$\frac{\sigma }{f_{rc}}=\left[\alpha \times (\varepsilon /\varepsilon_{rc)}+\left(3-2\alpha \right)\times {\left(\varepsilon /\varepsilon_{rc}\right)}^{2}+\left(\alpha -2\right){\left(\varepsilon /\varepsilon_{rc}\right)}^{3}\right],\;\left(\varepsilon \leq \varepsilon_{rc}\right)$
	$\frac{\sigma }{f_{rc}}=\frac{\varepsilon /\varepsilon_{rc}}{\beta \times {\left(\varepsilon /\varepsilon_{rc}-1\right)}^{2}+\varepsilon /\varepsilon_{rc}},\;\left(\varepsilon \geq \varepsilon_{rc}\right)$
	$\alpha =\left(2.4-0.0125f_{c}\right)\times k^{-2.195}\times \left(1-0.0027\rho d^{-0.1136}\right)$
	$\beta =\left(0.157f_{c}^{0.785}-0.905\right)\times exp\left(-0.1633ln\rho +\frac{0.22293k^{1.817\times 10^{11}}\rho^{-0.0434}d^{0.9924}}{d-0.0817}\right)$
	$f_{rc}=f_{c}\times exp\left(0.0222lnd-\frac{0.0054k^{-2.212}\rho^{1.088}d^{0.908}}{d-0.0175}\right)$
	$\varepsilon_{rc}=\varepsilon_{c}\times exp\left(0.31088ln\rho -\frac{0.3365\rho^{0.3931}d^{0.934}}{d+0.0441}\right)$
Aslani [21]	$\frac{\sigma }{f_{rc}}=\left[\frac{\rho_{m}\left(\varepsilon /\varepsilon_{rc}\right)}{\rho_{m}-1+{\left(\varepsilon /\varepsilon_{rc}\right)}^{\rho_{m}}}\right]$
	$f_{rc}=f_{c}\cdot \alpha \cdot e^{\left(-\beta \cdot R\right)}$
	$\rho_{m,a}={\left[1.02-1.17\left(E_{p}/E_{rc}\right)\right]}^{-0.74},\;\left(\varepsilon \leq \varepsilon_{rc}\right)$
	$\rho_{m,d}=\rho_{m}+\left(\varphi +\kappa \right),\;\left(\varepsilon \geq \varepsilon_{rc}\right)$
	$\varphi =35\times {\left(12.4-1.66\times 10^{-2}f_{rc}\right)}^{-0.9}$
	$\kappa =0.75\exp\left(-\frac{911}{f_{rc}}\right)$
	$E_{p}=f_{rc}/\varepsilon_{rc}$
	$\varepsilon_{rc}=\left(f_{rc}/E_{rc}\right)\left(\frac{v}{v-1}\right)$
	$E_{rc}=E_{c}\cdot \varphi \cdot e^{\left(-\psi \cdot R\right)}$
	$v=f_{rc}/17+0.8$
Bompa et al. [3]	$\sigma =E_{rc}\varepsilon_{rc,1}$,⋯$\varepsilon_{rc}\leq \varepsilon_{rc,el}$
	$\frac{\sigma }{f_{rc}}=\left[\frac{5}{3}\times \left(\frac{\varepsilon -\varepsilon_{rc,el}}{\varepsilon_{rc}}\right)-{\left(\frac{\varepsilon -\varepsilon_{rc,el}}{\varepsilon_{rc}}\right)}^{2}+\frac{0.3f_{rc}}{f_{rc}}\right],\;\;\varepsilon \;\epsilon \left(\varepsilon_{rc,el},\;\varepsilon_{cr}\right)$
	$\frac{\sigma }{f_{rc}}=\left[\frac{1}{8}\times \left(\frac{f_{rc}}{g_{c,2}}-1\right){\left(\frac{\varepsilon -\varepsilon_{rc1,2}}{\varepsilon_{rc}}\right)}^{2}-\frac{6}{8}\left(\frac{f_{rc}}{g_{c,2}}-1\right)\left(\frac{\varepsilon -\varepsilon_{rc1,2}}{\varepsilon_{rc}}\right)+\frac{f_{rc,2}}{f_{rc}}\right],\;\;\varepsilon \;\epsilon \left(\varepsilon \geq \varepsilon_{rc}\right)$
	$\frac{f_{rc,2}}{f_{rc}}=\left[\frac{5}{3}\times \left(\frac{\varepsilon_{rc}-\varepsilon_{rc,el}}{\varepsilon_{rc}}\right)-{\left(\frac{\varepsilon_{rc}-\varepsilon_{rc,el}}{\varepsilon_{rc}}\right)}^{2}+\frac{0.3f_{rc}}{f_{rc}}\right],\;\;$
	$f_{rc}=\frac{1}{1+2{\left(\frac{3\lambda \rho_{vr}}{2}\right)}^{3/2}}f_{c}$
	$\lambda =\{\begin{array}{c} 2.43\to \;d_{g,repl}\;\epsilon \;\left(0,5\right)\\ 2.90\to \;d_{g,repl}\;\epsilon \;\left(0,d_{g,max}\right)\\ 2.08\to \;\;d_{g,repl}\;\epsilon \;\left(5,d_{g,max}\right) \end{array}$
	$E_{rc}=12{\left(f_{rc}/10\right)}^{2/3}$
	$\varepsilon_{rc,el}=0.3f_{rc}/E_{rc}$
	$\varepsilon_{rc1,2}=\frac{4}{3\left(1-\rho_{vr}\right)}\varepsilon_{rc}$

**Table 3:** Concrete mixes design.

#	Mix ID	w/c	Cement 32.5 N [kg/m^3^]	Water [kg/m^3^]	GL ACE 430 [kg/m^3^]	RM [kg/m^3^]	CR 0–4 mm [kg/m^3^]	MA 0–2 mm [kg/m^3^]	MA 0–4 mm [kg/m^3^]	MA 4–8 mm [kg/m^3^]	MA 8–16 mm [kg/m^3^]	Add. Water [kg/m^3^]
Series I	NC-R0-400	0.45	400	180.0	2.0	0.0	0.0	323.6	554.4	288.4	630.6	48.0
	NC-R10-400	0.45	400	180.0	2.0	0.0	66.8	238.6	462.0	288.4	630.6	44.6
	NC-R20-400	0.45	400	180.0	2.0	0.0	133.6	153.4	369.6	288.4	630.6	41.2
	NC-R30-400	0.45	400	180.0	2.0	0.0	200.6	68.2	462.0	288.4	630.6	41.4
	NC-R40-400	0.45	400	180.0	4.0	0.8	266.2	0.0	166.4	288.4	630.6	34.4
Series II	NC-R0-500	0.45	500	198.1	2.6	0.0	0.0	281.0	484.6	252.0	551.4	0.0
	NC-R10-500	0.45	500	225.0	2.6	0.0	58.4	208.6	403.8	252.0	551.4	0.0
	NC-R20-500	0.45	500	225.0	2.6	0.0	116.8	134.0	323.0	252.0	551.4	0.0
	NC-R30-500	0.45	500	225.0	2.6	0.0	175.2	59.6	242.4	252.0	551.4	0.0
	NC-R40-500	0.45	500	225.0	2.6	0.0	233.8	0.0	145.4	252.0	551.4	30.0

**Table 4:** Results of fresh concrete properties.

#	Mix ID	Mass [kg]		Density		Porosity [%]		Slump	
Series I	NC-R0-400	18.64	REF	2330	REF	2.5	REF	19.50	REF
	NC-R10-400	17.97	−3.59%	2246	−3.61%	3.5	+40.00%	22.50	+15.38%
	NC-R20-400	16.76	−10.08%	2095	−10.09%	4.0	+60.00%	18.00	−7.69%
	NC-R30-400	15.89	−14.75%	1986	−14.76%	6.6	+164.00%	12.00	−38.46%
	NC-R40-400	15.51	−16.79%	1938	−16.82%	7.2	+188.00%	16.50	−15.38%
Series II	NC-R0-500	18.84	REF	2355	REF	1.4	REF	21.50	REF
	NC-R10-500	18.06	−4.14%	2257	−4.16%	1.8	+28.57%	20.00	−6.98%
	NC-R20-500	17.29	−8.22%	2161	−8.24%	2.5	+78.57%	20.00	−6.98%
	NC-R30-500	16.47	−12.58%	2059	−12.57%	3.0	+114.28%	21.50	0
	NC-R40-500	15.64	−16.98%	1955	−16.99%	5.1	+264.28%	11.00	−48.48%

**Table 5:** Compressive strength, elastic modulus and stress-strain test results.

#	Mix ID	Results Obtained on Cubic Specimens			Results Obtained on Cylindrical Specimens				
		$\mathrm{Compressive}\;\mathrm{Strength}\;f_{c}\;$			$\mathrm{Modulus}\;\mathrm{of}\;\mathrm{Elasticity}\;E_{c}\;$	Peak Compressive Stress s_max_			Deformation at Peak Compressive Stress e
		$\mathrm{Mean}\;f_{c}\;[\mathrm{MPa}]$	St. dev. [MPa]	CoV [%]	$\mathrm{Mean}\;E_{c}\;[\mathrm{MPa}]$	Mean s_max_ [MPa]	St. dev. [MPa]	CoV [%]	Mean e
Series I	NC-R0-400	29.06	1.42	4.88	39648.67	38.12	9.14	27.17	0.0029
	NC-R10-400	22.58	0.32	1.39	38153.00	22.52	2.22	10.42	0.0035
	NC-R20-400	14.05	0.59	4.21	28991.67	11.03	1.35	14.19	0.0052
	NC-R30-400	11.02	0.65	5.86	13101.33	8.14	1.37	16.84	0.0040
	NC-R40-400	3.75	0.97	25.85	3895.00	2.99	0.05	1.67	0.0078
Series II	NC-R0-500	42.66	0.93	2.18	40008.00	49.95	1.33	2.66	0.0036
	NC-R10-500	35.79	0.99	2.78	30183.00	31.89	1.39	4.37	0.0044
	NC-R20-500	23.88	0.91	3.79	24808.33	22.38	2.11	9.45	0.0058
	NC-R30-500	15.24	0.58	3.83	18349.00	12.77	0.49	3.88	0.0045
	NC-R40-500	10.43	0.97	9.3	9245.33	8.48	0.72	8.49	0.0052

**Table 6:** Comparison of experimentally and analytically obtained results of peak stress $f_{rc}$ and strain $\varepsilon_{rc}$ of rubberized concrete.

Mix ID	Experimentally Obtained Results		Analytically Obtained Results							
			L. Li et al. [18]		Aslani [20]		Bompa et al. [3]		Improved Model	
	$f_{rc}$ $\left[N/mm^{2}\right]$	$\varepsilon_{rc}$ [mm/mm]	$f_{rc}$ $\left[N/mm^{2}\right]$	$\varepsilon_{rc}$ [mm/mm]	$f_{rc}$ $\left[N/mm^{2}\right]\;$	$\varepsilon_{rc}$ [mm/mm]	$f_{rc}$ $\left[N/mm^{2}\right]$	$\varepsilon_{rc}$ [mm/mm]	$f_{rc}$ $\left[N/mm^{2}\right]$	$\varepsilon_{rc}$ [mm/mm]
NC-R10-500	31.89	0.0044	25.22 (−6.67)	0.0028 (−0.0016)	31.91 (+0.02)	0.0038 (−0.0006)	29.44 (−2.45)	0.0026 (−0.0018)	**29.56 (−2.33)**	**0.0040 (−0.0004)**
NC-R20-500	22.38	0.0058	9.79 (−12.59)	0.0020 (−0.0038)	25.87 (+3.49)	0.0038 (−0.0020)	18.89 (−3.49)	0.0027 (−0.0031)	**18.93 (−3.45)**	**0.0042 (−0.0016)**
NC-R30-500	12.77	0.0045	1.94 (−10.83)	0.0016 (−0.0029)	20.99 (+8.22)	0.0040 (−0.0005)	12.90 (+0.23)	0.0030 (−0.0015)	**12.98 (+0.21)**	**0.0047 (+0.0002)**
NC-R40-500	8.48	0.0052	0.11 (−8.37)	0.0014 (−0.0038)	16.99 (+8.51)	0.0042 (−0.0010)	9.44 (+0.96)	0.0037 (−0.0013)	**9.44 (+0.94)**	**0.0058 (+0.0006)**

**Table 7:** Statistical performance measures.

Statistical Parameter	Equation		Statistical Parameter	Equation	
MAE	$\frac{1}{n}\sum_{i=1}^{n}\left|y-y{}^{\prime }\right|$	(10)	MAPE	$\frac{1}{n}\sum_{i=1}^{n}\left|\frac{y-y^{\prime }}{y}\right|$	(12)
RMSE	$\sqrt{\frac{1}{n}\sum_{i=1}^{n}{\left(y^{\prime }-y\right)}^{2}}$	(11)	R	$\frac{\sum_{i=1}^{n}\left(\left(y-\bar{y}\right)\cdot \left(y^{\prime }-\bar{y}{}^{\prime }\right)\right)}{\sqrt{\sum_{i=1}^{n}{\left(y-\bar{y}\right)}^{2}\cdot \sum_{i=1}^{n}\left(y{}^{\prime }-\bar{y}{}^{\prime }\right)}}$	(13)
	*y’* presents the modelled value of compressive strength; *y* is the experimental value; and *n* is the number of data samples, y’ is the mean modelled value; *y* is the mean experimental value.				

**Table 8:** Statistical performance of Constitutive models for experimental study.

Mix ID	MAE (10) [MPa]	RMSE (11) [MPa]	MAPE (12) [%]	R (13)
NC-R10-500	2.60	1.58	16.56	0.96
NC-R20-500	5.52	1.82	27.91	0.77
NC-R30-500	1.40	0.93	18.97	0.88
NC-R40-500	0.77	0.36	13.97	0.92

**Table 9:** Statistical performance of Constitutive models for validation samples.

Mix ID	MAE (10) [MPa]	RMSE (11) [MPa]	MAPE (12) [%]	R (13)
L.Li-6% [18]	2.51	0.62	14.47	0.87
L.Li-8% [18]	1.95	0.39	12.74	0.90
L.Li-10% [18]	3.37	1.61	23.56	0.90
Bompa- 0% [3]	1.10	2.41	24.53	0.84
Bompa 20% [3]	3.68	0.37	30.27	0.89

## References

[1] Sabău M., Oneţ T. (2011). Nonlinear concrete behaviour. J. Appl. Eng. Sci..

[2] Park R. (1991). Ductility of structural concrete. IABSE Rep..

[3] Bompa D.V., Elghazouli A.Y., Xu B., Stafford P.J., Ruiz-Teran A.M. (2017). Experimental assessment and constitutive modelling of rubberised concrete materials. Constr. Build. Mater..

[4] Khaloo A.R., Dehestani M., Rahmatabadi P. (2008). Mechanical properties of concrete containing a high volume of tire-rubber particles. Waste Manag..

[5] Ismail M.K., Hassan A.A.A. (2016). Performance of full-scale self-consolidating rubberized concrete beams in flexure. ACI Mater. J..

[6] Wang P.T., Shah S.P., Naaman A.E. (1978). Stress-Strain Curves of Normal and Lightweight Concrete in Compression. ACI J. Proc..

[7] Van Gysel A., Taerwe L. (1996). Analytical formulation of the complete stress-strain curve for high strength concrete. Mater. Struct..

[8] Wee T.H., Chin M.S., Mansur M.A. (1996). Stress-Strain Relationship of High-Strength Concrete in Compression. J. Mater. Civ. Eng..

[9] Chin M.S., Wee T.H., Mansur M.A. (1995). Derivation of the Complete Stress–Strain Curves for Concrete in Compression. Mag. Concr. Res..

[10] Carreira D.J., Chu K.-H. (1985). Stress-Strain Relationship for Plain Concrete in Compression. ACI J..

[11] Lee I. (2002). Complete Stress-Strain Characteristics of High Performance Concrete. Dissertations. https://digitalcommons.njit.edu/dissertations/532.

[12] Kristiawan S.A. (2018). Uniaxial Compressive Stress-Strain Behavior of Self-Compacting Concrete with High-Volume Fly Ash. Int. J. Geomate.

[13] Kristiawan S., Sunarmasto A.S., Budi A.S., Kurniawati D.C. Stress-strain response of high-volume fly ash self-compacting concrete (HVFA-SCC) under uniaxial loading and its effect on the reinforced HVFA-SCC nominal strength. Proceedings of the 4th International Conference on Rehabilitation and Maintenance in Civil Engineering.

[14] Hognestad E. (1951). Study of Combined Bending and Axial Load in Reinforced Concrete es.

[15] Kent D.C., Park R. (1971). Flexural members with confined concrete. J. Struct. Div..

[16] Mander J.B., Priestley M.J.N., Park R. (1988). Theoretical stress-strain model for confined concrete. ASCE J. Struct. Eng..

[17] Comité Euro-International du Béton (CEB) (1990). CEB Model. Code 1990.

[18] Li L., Ruan S., Zeng L. (2014). Mechanical properties and constitutive equations of concrete containing a low volume of tire rubber particles. Constr. Build. Mater..

[19] Xie J.H., Guo Y.C., Liu L.S., Xie Z.H. (2015). Compressive and flexural behaviours of a new steel-fibre-reinforced recycled aggregate concrete with crumb rubber. Constr. Build. Mater..

[20] Aslani F. (2016). Mechanical Properties of Waste Tire Rubber Concrete. J. Mater. Civ. Eng..

[21] Aslani F., Ma G., Yim Wan D.L., Tran Le V.X. (2018). Experimental investigation into rubber granules and their effects on the fresh and hardened properties of self-compacting concrete. J. Clean. Prod..

[22] Aslani F., Ma G., Yim Wan D.L., Muselin G. (2018). Development of high-performance self-compacting concrete using waste recycled concrete aggregates and rubber granules. J. Clean. Prod..

[23] Li D., Zhuge Y., Gravina R., Mills J.E. (2018). Compressive stress strain behavior of crumb rubber concrete (CRC) and application in reinforced CRC slab. Constr. Build. Mater..

[24] Mendis A.S., Al-Deen S., Ashraf M. (2017). Behaviour of similar strength crumbed rubber concrete (CRC) mixes with different mix proportions. Constr. Build. Mater..

[25] Noaman A.T., Abu Bakar B.H., Akil H.M. (2016). Experimental investigation on compression toughness of rubberized steel fibre concrete. Constr. Build. Mater..

[26] Guo Y.C., Zhang J.H., Chen G.M., Xie Z.H. (2014). Compressive behaviour of concrete structures incorporating recycled concrete aggregates, rubber crumb and reinforced with steel fibre, subjected to elevated temperatures. J. Clean. Prod..

[27] Gupta T., Chaudhary S., Sharma R.K. (2014). Assessment of mechanical and durability properties of concrete containing waste rubber tire as fine aggregate. Constr. Build. Mater..

[28] Hilal N.N. (2017). Hardened properties of self-compacting concrete with different crumb rubber size and content. Int. J. Sustain. Built Environ..

[29] Turatsinze A., Garros M. (2008). On the modulus of elasticity and strain capacity of Self-Compacting Concrete incorporating rubber aggregates. Resour. Conserv. Recycl..

[30] Babu R.R., Benipal G.S., Singh A.K. (2005). Constitutive modelling of concrete: An overview. Asian J. Civ. Eng. (Build. Hous.).

[31] Code for Design of Concrete Structures—Chinese Code GB 50010–52010. https://dokumen.tips/documents/code-for-design-of-concrete-structures-chinese-code-gb-50010-2010.html.

[32] Aslani F., Nejadi S. (2012). Cyclic constitutive model for high-strength concrete confined by ultra-high-strength and normal-strength transverse reinforcements. Aust. J. Struct. Eng..

[33] (2009). HRN EN 12350-2:2009 Testing Fresh Concrete—Part 2: Slump-Test.

[34] (2009). HRN EN 12350-6:2009 Testing Fresh Concrete—Part 6: Density.

[35] (2009). HRN EN 12350-7:2009 Testing Fresh Concrete—Part 7: Air Content.

[36] (2012). HRN EN 12390-1:2012 Testing Hardened Concrete—Part 1: Shape, Dimensions and Other Requirements for Specimens and Moulds.

[37] (2009). HRN EN 12390-2:2009 Testing Hardened Concrete—Part 2: Making and Curing Specimens for Strength Tests.

[38] (2009). HRN EN 12390-3:2009 Testing Hardened Concrete—Part 3: Compressive Strength Test of Specimens.

[39] (2000). HRN EN 12390-4:2000 Testing Hardened Concrete—Part 4: Compressive Strength-Specifications for Testing Machines.

[40] (2009). HRN EN 12390-13:2013 Testing Hardened Concrete—Part 13: Determination of Secant Modulus of Elasticity in Compression.

[41] Bahr O., Schaumann B., Bollen B., Bracke J. (2013). Young’s modulus of Poisson’s ratio of concrete at high temperatures: Experimental investigations. Mater. Des..

